# Systematic Review Protocol for the Current State of Chemical Exposure in Infants via Breast Milk, Artificial Milk and Dairy Products

**DOI:** 10.3390/ijerph18094436

**Published:** 2021-04-22

**Authors:** Manal A. M. Mahmoud, Hosnia Abdel-Mohsein, Usama Mahmoud, Zhaoqing Lyu, Sani Rachman Soleman, Meng Li, Tomoko Fujitani, Mariko Harada Sassa, Yukiko Fujii, Yang Cao, Toshiaki Hitomi, Kouji H. Harada

**Affiliations:** 1 Department of Animal Hygiene, Faculty of Veterinary Medicine, Assiut University, Assiut 71526, Egypt; hosnia18@aun.edu.eg; 2 Department of Animal and Poultry Behavior and Management, Faculty of Veterinary Medicine, Assiut University, Assiut 71526, Egypt; usama.mahmoud@aun.edu.eg; 3 Department of Health and Environmental Sciences, Kyoto University Graduate School of Medicine, Kyoto 606-8501, Japan; lzq_1726362531@yahoo.co.jp (Z.L.); sani.rachman@uii.ac.id (S.R.S.); lime_366@yahoo.co.jp (M.L.); fujitani.tomoko.4w@kyoto-u.ac.jp (T.F.); marikohs@kuhp.kyoto-u.ac.jp (M.H.S.); 4 Department of Public Health, Faculty of Medicine, Universitas Islam Indonesia, Yogyakarta 55584, Indonesia; 5 Department of Pharmaceutical Sciences, Daiichi University of Pharmacy, Fukuoka 815-8511, Japan; yu-fujii@umin.ac.jp; 6 Department of Preventive Medicine, St. Marianna University School of Medicine, Kawasaki 216-8511, Japan; soyo_cao@hotmail.com (Y.C.); thitomi@marianna-u.ac.jp (T.H.)

**Keywords:** systematic review, environmental pollutants, infants, breastfeeding, artificial formula

## Abstract

Many studies have shown that human breast milk is contaminated with various chemicals. In the proposed systematic review, the aim is to identify and summarize the available literature regarding chemical exposure via breastfeeding or the feeding of artificial formula. MEDLINE (PubMed) will be the primary source in this literature search. Primary studies that analyzed one or more chemicals of interest in breast milk or artificial milk and that reported information on concentrations will be eligible for this review. Conference abstracts will not be included in the review unless access to the data is easy. First, the titles and abstracts of identified articles will be screened by two or more researchers. Then, a full-text review will be conducted to extract data from the included articles and code them for classification. The results of the search and classification will be summarized narratively and bibliometrically. The aim of the review is to analyze trends in publications according to year and region from the viewpoint of target chemicals, location, range of concentrations, and health outcomes.

## 1. Introduction

Breastfeeding is the standard in infant nutrition. “Infant” is a term which is used for a very young offspring until 12 months of age. However, it may cause anxiety for mothers who have difficulty breastfeeding and may place a burden on the mother’s lifestyle and behavior. Thus, feeding artificial milk is an alternative to breastfeeding. The American Academy of Pediatrics recommends breastfeeding for about six months and continuing breastfeeding for more than one year, if possible [1]. Advantages of breast milk have been proven with respect to nutrition, immunological components, and neurodevelopmental benefits. Breastfeeding is estimated to reduce future overweight and obesity by 13% [2]. Breastfed children are estimated to have an intelligence quotient approximately three points higher than babies fed with artificial milk [3]. Hence, infant nutrition is an important public health issue.

With the developments in the chemical industry, many organic compounds have been synthesized and introduced into common use. These chemicals have improved people’s lives and provide various conveniences. For example, owing to their flame-retardant characteristics, polychlorinated biphenyls (PCBs) are used for insulating oils in condensers and as heat transfer media, as well as solvents in carbonless copy paper, among other uses. Dichlorodiphenyltrichloroethane, known as DDT, and hexachlorocyclohexane, are inexpensive insecticides that have been used for pest control. These organic compounds were initially called “miracle chemicals” and contributed to economic growth. However, it has become clear that these chemicals persist in the environment owing to their chemical stability and can cause adverse effects in living organisms.

Many studies have shown that human breast milk is contaminated with such chemicals. PCBs, DDT, and other chemicals are fat-soluble and can be transferred to breast milk from blood [4]. Therefore, breastfed infants are exposed to these chemicals even in a short period. Since 1976, the World Health Organization has conducted surveys of breast milk because the matrix is easy for monitoring fat-soluble contaminants and suitable for assessing the exposure of mothers and infants [5]. In recent years, endocrine-disrupting chemicals with estrogen-like effects detected in breast milk have posed concerns [6]. Artificial milk and dairy products are industrially produced using cow’s milk as a basic raw material. Cow’s milk has a composition similar to human breast milk and contains high levels of fat; thus, fat-soluble substances can easily accumulate in cow’s milk. In fact, organochlorine insecticides have been detected in dairy products [7,8]. This suggests that chemicals in the environment and feed for dairy cows are transferred to milk, with levels and patterns that vary according to the type and amount of chemicals used in the place of origin. Therefore, exposure to these chemicals may occur from both breast milk and artificial milk.

Exposure assessments of various chemicals have been conducted, but the target compounds are constantly changing. Organochlorine compounds were commonly used for decades. Among organohalogen compounds, brominated compounds have been used as flame retardants [9,10,11]; some of them have a structure similar to that of naturally occurring chemicals [12]. In addition, organofluorine compounds such as per- and polyfluoroalkyl substances (PFAS), used as water repellents and surfactants, emerged after 2000 [13]. Organohalogen compounds are not limited to the above but are diverse and have been detected in breast milk [14,15]. In addition to persistent pollutants, chemicals with short half-lives were detected in breastmilk, such as bisphenols [16,17], phthalate esters [18], and UV-filter benzophenones [19]. Pharmaceutical and personal care products (PPCPs) can also be transferred from maternal blood to breast milk during the medication of mothers [20]. In artificial milk, the effects of veterinary medicines (antibiotics, growth hormone agents, etc.) administered to dairy cows should also be considered [21,22]. Radionuclides are another concern in nuclear disasters, which contaminate breast milk and artificial milk [23,24]. Environmental pollution with these compounds can manifest as different aspects within the socioeconomic and industrial context of each region. The methods to investigate chemical exposure in children from breast milk or artificial milk have not been consistent. There are no comprehensive reviews available to help clarify the current status of chemical exposure from both breast milk and artificial milk. 

The purposes of the proposed review are: (1) to identify monitoring studies of breast milk and artificial milk so as to assess chemical exposure among infants; (2) to describe studies with respect to the target population, region, and chemicals; (3) to summarize potential health risks according to studies reporting an association between chemical exposure and health outcomes; and (4) to consider the current knowledge, evidence gaps, and necessary future investigations. Therefore, a protocol for a systematic review of studies using a matrix of breast milk and artificial milk is presented. This systematic review is expected to identify evidence gaps and the existence of evidence clusters that can be used to develop future research questions.

## 2. Materials and Methods

### 2.1. Data Source

The aim of this review is to identify and summarize available scientific studies measuring environmental pollutants in human breast milk or artificial milk (including raw milk and dairy products). Published, peer-reviewed studies will be identified by searching the MEDLINE (PubMed) electronic database, with no language restrictions. Searching will also be conducted in databases such as Web of Science and Embase; however, MEDLINE will be the primary data source. This study will mainly aim to evaluate current research trends; therefore, the period of literature surveyed will be limited to within approximately the past 10 to 30 years, according to the number of available studies and/or their history of applications. For example, classical contaminants such as PCBs and DDTs have been used over several decades, but radionuclides could have been investigated in rare events (e.g., Chernobyl and Fukushima accidents). The publication period will be limited using the PubMed Advanced Search Builder with the Create Date field. In addition to publications identified in the database search, references contained in the identified literature and related reviews may also be referenced.

The search terms to be used in the MEDLINE search are shown in Table 1. The search results will be narrowed down using search terms to identify the target chemical substances. There will be no restriction for search results regarding health outcomes.

### 2.2. Eligibility Criteria

Original articles analyzing one or more chemicals of interest in breast milk and/or artificial milk and reporting information on concentrations are eligible. Studies that do not contain such information will be excluded by screening titles and abstracts. Appropriate studies in languages other than English will be included as much as possible. Conference proceedings will not be included in the review unless the detailed content is easily accessible.

### 2.3. Management of Literature 

The literature search results will be imported into platforms such as EndNote and Mendeley. Sharing of the data among investigators will be facilitated using cloud services.

### 2.4. Literature Screening

The title and abstract of the publications identified in the initial search will be screened by two or more investigators. SWIFT ActiveScreener software or similar software will be used for this process. To maximize the number of included publications, more than half of the investigators must agree to the exclusion of any article. One-quarter of the researchers must agree for study inclusion. For example, if only two researchers are making the determination, one includes a study and another rejects it, the study will be included. The full text of publications included after primary screening will be further reviewed. 

In the full-text review, data contained in each article will be extracted and coded for classification. In addition to primary reviewers, secondary reviewers will review and supervise data extraction and coding. For unclear information, there is an option to annotate and, if possible, contact the original author.

### 2.5. Data Coding

Data extraction will be performed using the form in Table 2. Author information, journal title, publication year, target population, target chemicals, analytical methods and detection limits, and health outcomes (if any) are to be collected. To consider potential differences in exposure between countries, background information such as ethnicity, body weight at sampling, birth weight, and lactation period will also be extracted if they are available. For studies that include health outcomes, research design and outcome indicators will also be extracted.

### 2.6. Evaluation of the Quality of Research

There is an option to annotate the quality of the study as to whether the population, analytical methods, detection limits, etc., are appropriate.

### 2.7. Summary of Results

The results of the literature search and classification are to be summarized narratively and quantitatively (Table 3). It is also aimed to analyze trends in research publications according to period and region, and from the viewpoints of target chemicals, study location, exposure levels, and health outcomes.

### 2.8. Registration of Individual Protocols

Systematic review protocols for each target group will be registered in PROSPERO (https://www.crd.york.ac.uk/prospero/, accessed on 1 April 2021) [25].

## 3. Conclusions

This systematic review protocol has been developed to identify evidence gaps and the future research needed regarding chemical contamination in breast milk and artificial milk.

## Figures and Tables

**Table 1:** Search strategy in the proposed review.

Databases	Pubmed (MEDLINE), (Optional: Web of Science, Embase, CINAHL, and SciFinder Scholar)
PubMed search terms:	
for breast milk	(“breast milk *” OR “mother’s milk *” OR “mothers’ milk *” OR “human milk *”) AND (“level *” OR “concentration *” OR “exposure *” OR “monitor *” OR “occurrence” OR “detect *” OR “measure *” OR “determin *”)
for artificial milk	(“dairy product *” OR “formula” OR “baby milk *” OR “infant milk *” OR “artificial milk *”) AND (“level *” OR “concentration *” OR “exposure *” OR “monitor *” OR “occurrence” OR “detect *” OR “measure *” OR “determin *”)
for animal milk	(“cow milk *” OR “ewe milk *” OR “goat milk *” OR “buffalo milk *”) AND (“level *” OR “concentration *” OR “exposure *” OR “monitor *” OR “occurrence” OR “detect *” OR “measure *” OR “determin *”)
for dairy products	(“butter *” OR “cheese *” OR “cream *” OR “yogurt *” OR “ghee *” OR “condensed milk *” OR “dried milk *” OR “ice cream *” OR “dairy”) AND (“level *” OR “concentration *” OR “exposure *” OR “monitor *” OR “occurrence” OR “detect *” OR “measure *” OR “determin *”)
Target compounds	Results will be screened further using specific keywords
for organohalogen compounds	(“chlor *” OR “organochlor *” OR “brom *” OR “organobrom *” OR “fluor *” OR “perfluor *” OR “polyfluor *” OR “halog *” OR “organohalo *”)
for chemicals with short halflives	(“phthalate *” OR “plasticizer *” OR “paraben *” OR “bisphenol *” OR “benzophenone *” OR “UV filter *”)
for antibiotics	(“tetrac *” OR “norfl *” OR “cipro *” OR “tylosin” OR “* lactam” OR “mastitis” OR “bacteri *” OR “resist *”)
for general contaminants and PPCPs	(“pollut *” OR “contam *” OR “drug *” OR “medicin *” OR “pharmaceut *” OR “personal care *”)
Search period	Latest 10–30 years (e.g., December 2010 to December 2020) or longer
Inclusion criteria	Analysis of contaminants using quantitative methods; analysis of target specimens; analysis of specified target contaminants
Exclusion criteria	Review paper; opinions; conference proceedings (unless access to the data is easy)

The asterisk (*) indicates wildcards in search terms.

**Table 2:** Summary of data items to be extracted.

Category	Items
Study location	Country, area, city, etc.
Year of study	Sampling periods
Study design	Cross-sectional, cohort, single center; sampling method, etc.
Number of participants	Pooled samples or individual samples
Population data	Mother’s age, parity, child’s age in months, ethnicity, body weight at sampling, birth weight, lactation period, etc.
Specimen type	Breast milk, artificial milk, animal milk and dairy products
Chemical group	Example: PCB, DDT, PFAS, antibiotics, growth hormone, etc.
Summary of results	Average concentration, median, range; daily intake; hazard index, margin of exposure; factors affecting exposure; health outcomes, etc.

**Table 3:** Output of systematic review and synthesis of the results.

Summary Statistics	Items
Publication trends	Number, location, target chemicals
Study designs and endpoints	Concentration; daily intake; hazard index; epidemiology, etc.
Levels and exposures to pollutants, by location	Average, median, range, etc.
Health outcomes	Endpoints; odds ratio; risk ratio; margin of exposure, etc.

## Data Availability

Not applicable.
